# Methodological issues in estimating sodium intake in the Korea National Health and Nutrition Examination Survey

**DOI:** 10.4178/epih/e2014033

**Published:** 2014-11-28

**Authors:** Hyun Ja Kim, Kyungwon Oh

**Affiliations:** Division of Health and Nutrition Survey, Korea Centers for Disease Control and Prevention, Cheongju, Korea

**Keywords:** Sodium, Urine, Nutrition assessment, Dietary Reference Intake, Validity, Korea National Health and Nutrition Examination Survey

## Abstract

For policy goal setting, efficacy evaluations, and the development of related programs for reducing sodium intake, it is essential to accurately identify the amount of sodium intake in South Korea and constantly monitor its trends. The present study aimed to identify the status of sodium intake in South Korea and to review the methods and their validity for estimating sodium intake in each country; through this, we aim to determine more accurate methods for determining sodium intake and to monitor the trend in sodium intake for Korean citizens in the future. Using 24-hour dietary recall data from the 2012 Korea National Health and Nutrition Examination Survey (KNHANES) to estimate daily sodium intake, the average daily sodium intake among Koreans was 4,546 mg (men, 5,212 mg; women, 3,868 mg). In addition to the nutrition survey that uses the 24-hour dietary recall method, sodium intake can also be calculated from the amount of sodium excreted in 24-hour urine, 8-hour overnight urine, and spot urine samples. Although KNHANES uses the 24-hour dietary recall method to estimate the sodium intake, the 24-hour dietary recall method has the disadvantage of not being able to accurately determine the amount of sodium intake owing to its unique characteristics of the research method and in the processing of data. Although measuring the amount of sodium excreted in 24-hour urine is known to be the most accurate method, because collecting 24-hour urine from the general population is difficult, using spot urine samples to estimate sodium intake has been suggested to be useful for examining the trend of sodium intake in the general population. Therefore, we planned to conduct a study for estimating of 24-hour sodium excretion from spot urine and 8-hour overnight urine samples and testing the validity among subsamples in the KNHANES. Based on this result, we will adopt the most appropriate urine collection method for estimating population sodium intake in South Korea.

## INTRODUCTION

Excessive sodium intake is a risk factor for hypertension, cardiovascular diseases, kidney diseases, and gastric cancer, and indirectly functions as a factor that can increase the risks of obesity, kidney stones, and osteoporosis. Reducing sodium intake significantly decreases the prevalence and mortality rates of chronic diseases [1]. It is well known that Korean consume too much sodium from traditional foods, such as kimchi, soy sauce and paste, salt-fermented seafood, soups, and stews; hence, policy-based approaches that can reduce sodium intake are urgently needed. Furthermore, in order to establish reduction goals and evaluate the effectiveness of sodium reduction programs, it is essential to accurately estimate sodium intake and continue to monitor trend in sodium intake in South Korea. Sodium intake can be investigated with nutrition surveys using 24-hour dietary recall or food records, as well as through amount of sodium excreted in 24-hour urine, 8-12 overnight urine, and spot urine samples; moreover, the methods for estimating sodium intake vary for each country, with each having its own advantages and disadvantages [2].

The aims of this study were to identify the status of sodium intake in South Korea and to review the methods for estimating sodium intake for each country and their validity; through this, we will establish a method for accurately estimating and monitoring sodium intake in a representative Korean population.

## STATUS OF SODIUM INTAKE IN SOUTH KOREA

**Figure 1.**
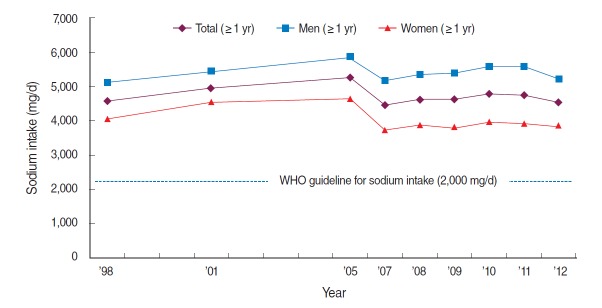
Trend in mean daily intake of sodium among Koreans above 1 year of age, Korea National Health and Nutrition Examination Survey (1998-2012). The age-standardized mean was calculated using an estimated population, based on the 2005 census of the Korean population. Since 2007, a revised food composition table was used to calculate the sodium intake.

According to the 2012 Korea National Health and Nutrition Examination Survey (KNHANES) [3], the daily sodium intake for Koreans was 4,546 mg (men, 5,212 mg; women, 3,868 mg), which exceeded 2-fold of the recommended maximum daily intake of 2,000 mg [4], established by the World Health Organization (WHO) (Figure 1). In looking at the trend in sodium intake (over 1 years old, age-standardized) since 1998, when KNHANES was first conducted, mean daily intake of sodium increased from 4,582 mg in 1998 to 5,260 mg in 2005; however, sodium intake decreased to 4,453 mg in 2007, when the revised food composition table was established. On the basis of the trends since 2007, wherein the same food composition table was applied, sodium intake showed slight increases at 4,608, 4,618, and 4,785 mg in 2008, 2009, and 2010, respectively, but it continuously decreased thereafter to 4,752 mg in 2011 and 4,546 mg in 2012. The daily sodium intake in men was higher than that in women by approximately 1,000-1,500 mg, which was attributable to higher consumption of food in men compared to women; however, annual trends for both men and women showed similar patterns.

**Figure 2.**
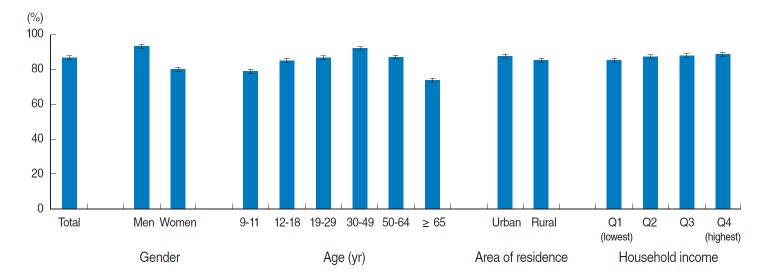
Prevalence of excessive sodium intake compared to the Korean Dietary Reference Intake among Koreans above 9 years of age, the 2012 Korea National Health and Nutrition Examination Survey. Values are presented as percentages and confidence intervals. The goal for sodium intake of Korean Dietary Reference Intake was established at 2,000 mg/d for those above 9 years of age. The age-standardized prevalence according to the area of residence and household income was calculated using an estimated population, based on the 2005 census of the Korean population.

In the 2012 KNHANES, the percentage of excessive sodium intake compared to the goal for sodium intake (9 years or older, 2,000 mg) established by the Korean Dietary Reference Intake was 93.3% for men and 79.8% for women aged 9 years or older (Figure 2); moreover, the percentage of excessive sodium intake in people in their 30s and 40s appeared to be the highest at 91.9% (men, 96.9%; women, 86.7%). Regardless of area of residence or household income level, over 85% of the subjects showed excessive sodium intake.

## ADVANTAGES, DISADVANTAGES, AND VALIDITY OF METHODS FOR ESTIMATING SODIUM INTAKE

Daily sodium intake can be measured with the nutrition survey methods, such as 24-hour dietary recall and food records, and through amount of sodium excreted in 24-hour urine, 8-12 overnight urine, and spot urine samples.

Among these methods, the 24-hour urinary sodium excretion is known to be the gold standard method for measuring sodium intake, because 85-95% of consumed sodium is excreted through urine and sodium in urine is highly correlated with sodium intake from food [5]. Nevertheless, the 24-hour urine collection has the disadvantages that urine collection without loss for 24-hour from the general population that engages in free lifestyle is difficult and imposes a high burden on the participants [2]. In the National Diet and Nutrition Survey (England), the North Karelia Salt Project (Finland), FINMONICA Study (Finland), and the national FINRISK Study (Finland), 24-hour urine from a representative sample has been continuously collected to monitor the average sodium intake of their citizens.

A nutrition survey using 24-hour dietary recall or food records can be a relatively useful method in a large-scale population study, but it is subject to several limitations [6]; there are difficulties in recalling exactly how much of what type food was consumed for the past 1 day, as well as determining exactly how much salt was added during cooking. In addition, by applying the same value of sodium in dish to all, it has the disadvantage of not being able to reflect individual differences in salt intake. Moreover, since the food composition table is amended every 5 years, it presents limitations in making comparisons in annual trends. Sodium intake has been estimated from a 24-hour dietary recall for 1-day in KNHANES, 24-hour dietary recall for 1-2 days in the National Health and Nutrition Examination Survey in the US, and a semi-weighted food record method for 1 day (3 days before 1995) in the Japanese National Nutrition Survey. The validity of 24-hour dietary recall methods for estimating sodium intake varies for each country, based on primary food consumed and cooking characteristics (Table 1) [7-11]. In the West, the primary source of sodium intake is processed foods (US, 77% [12]; UK, 65-70% [13]), which allows determination of a significant portion of individual sodium intake from food intake alone; thus, the correlation coefficients with 24-hour urinary sodium excretion were approximately 0.3-0.4 [7,9,11]. However, in a study on Koreans, the results indicated that the correlation coefficient between the sodium intake from 24-hour dietary recall and 24-hour urinary sodium excretion was 0.11 [10]. One of the reasons for such a low correlation is considered to be difficulties associated with the fact that the primary sources of sodium intake for Koreans are cooked dishes, such as kimchi, soups, and stews; the amount of ingredients and the amount of seasonings added could not be identified in the cooked foods, despite the fact that there can be significant differences among individuals based on sensitivity to salt and the amount of salt added during cooking. It makes difficult to estimate individuals’ sodium intake with the application of a standardized amount of ingredients in cooked dishes.

The use of sodium concentration in spot urine as a method for estimating daily sodium intake has the advantage of placing fewer burdens on participants, but it is limited in accurately measuring an individual’s sodium intake due to the fact that sodium concentration can easily be affected by the volume of fluid ingested [2]. Despite this disadvantage, spot urine has been considered a useful method for estimating population mean value of sodium intake. It makes it possible to compare the sodium intake between different population and monitor trends over time within a specific population [14]. The correlation coefficients between sodium concentration in spot urine and sodium concentration in 24-hour urine were 0.28-0.86 [14-19], with differences observed in the time and frequency of spot urine collection (Table 2). An 8-12 hour overnight urine method has the advantage of low-burden urine collection than 24-hour urine method, but it has the disadvantages of needing to collect the urine under strict time constraints and requires the assumption that urine excretion is constant day and night despite of usual diurnal pattern in excretion of sodium (i.e., higher excretion during the day). In the case of overnight urine, its correlation coefficients with 24-hour urine were 0.59-0.78 [20-22], which indicates that it is more valid as a substitute for 24-hour urine than spot urine (Table 2).

## IMPROVEMENT OF METHODS FOR ESTIMATING SODIUM INTAKE IN KOREA NATIONAL HEALTH AND NUTRITION EXAMINATION SURVEY

Although a 24-hour dietary recall method has been used to estimate the average sodium intake of Koreans in KNHANES, as previously mentioned, a 24-hour dietary recall method has limitation in making accurate measures of sodium intake owing to its unique characteristics as a research method and in the processing of data. As such, in order to improve upon the limitations of the nutrition survey, we planned to adopt methods for estimating sodium intake using urine in KNHANES; however, prior to this introduction, an examination of the feasibility and validity of various urine sodium measurement methods is required. Accordingly, a study on the estimation methods for sodium intake in KNHANES is currently underway as a research project of the Korea Centers for Disease Control and Prevention, and 24-hour urine, 8-hour overnight urine, and spot urine samples from 300 adults who had participated in KNHANES is collecting to develop a formula for estimating sodium intake for Korean, and to test the validity of the sodium intake estimation method using spot urine and 8-hour overnight urine samples through comparisons with 24-hour urinary sodium excretion. Through the present study, guidelines for the most appropriate urine collection method, collection time, and collection amount for estimating sodium intake of Koreans will be developed and adopted in KNHANES. It is anticipated that the sodium intake estimated with spot urine or 8-hour overnight urine samples will complement the results of the 24-hour dietary recall method used in KNHANES; moreover, the sodium intake by Koreans estimated through this method is expected to be used as the basis for promoting policies on sodium reduction, developing health policies, and evaluating their effectiveness.

## Figures and Tables

**Table 1.** Studies that compared the 24-hour dietary recall method with the 24-hour urine collection method for the estimation of sodium intake in a healthy population

Authors (published year) [Ref]	Country	Population	Sample size (age)	No. of days of 24-hour recall	No. of days of 24-hour urine	Correlation coefficient between dietary sodium intake and 24-hour urinary sodium
Espeland et al. (2001) [7]	US	Elderly cohort participants	M: 181, F: 160 (60-79 yr)	5	5	r = 0.30
Sasaki et al. (2003) [8]	Japan	Cohort participants	M: 32, F: 57	28	2	r = 0.38 for men
						r=0.47 for women
Reinivuo et al. (2006) [9]	Finland	Random population	M: 410, F: 469 (25-64 yr)	2	1	r = 0.3
Shin et al. (2010) [10]	South Korea	Healthy volunteers	F: 236 ( ≥ 20 yr)	3	1	r = 0.11
Rhodes et al. (2013) [11]	US	Healthy volunteers	M: 232, F: 233 (30-69 yr)	1-2	1-2	r = 0.18-0.43 according to the body mass index

Ref, reference; M, male; F, female.

**Table 2.** Studies that compared spot or overnight urine samples with the 24-hour urine samples for the estimation of sodium intake in a healthy population

Authors (published year) [Ref]	Country	Population	Sample size (age)	Type of urine sample	Correlation coefficient between spot or overnight urinary sodium and 24-hour urinary sodium
Spot urine samples					
Kawasaki et al. (1982) [15]	Japan	Healthy volunteers	M: 91, F: 151 (20-63 yr)	Spot urine	r = 0.47 for 1 d
					r = 0.73 for 1 d after discarding outliers
					r = 0.62 for 3 d
Kawasaki et al. (1993) [16]	Japan	Healthy free-living individuals	Adults: 159 (20-79 yr)	Spot urine (second morning urine)	r = 0.728
					r = 0.51 for 1 d to 0.82 for 3 d
Costa et al. (1994) [17]	Brazil	Healthy individuals	Adults: 611 (20-74 yr)	Spot urine	r = 0.28
Tanaka et al. (2002) [14]	Japan	INTERSALT participants	M: 295, F: 296 (20-59 yr)	Spot urine	r = 0.54
Mann & Gerber (2010) [18]	US	Unselected volunteers	M: 81 (21-82 yr)	Spot urine	r = 0.17
				Morning urine	r = 0.31
				Evening urine	r = 0.86
Brown et al. (2013) [19]	Europe	INTERSALT participants (29 populations)	M: 1,369, F: 1,376 (20-59 yr)	Spot urine (individual level)	r = 0.50 for men
				Spot urine (population level)	r = 0.51 for women
					r = 0.79 for men
					r = 0.71 for women
Overnight urine samples					
Watson & Langford (1970) [20]	US	Students	F: 52 (20-22 yr)	Overnight urine	r = 0.76
Liu et al. (1979) [21]	US	Volunteers	M: 116 (30-44 yr)	Overnight urine	r = 0.72
Kamata & Tochikubo (2002) [22]	Japan	Healthy individuals	M: 71. F: 78 (35-49 yr)	Overnight urine	r = 0.73 for men
					r = 0.78 for women

Ref, reference; M, male; F, female.

## References

[1] He FJ, MacGregor GA (2010). Reducing population salt intake worldwide: from evidence to implementation. Prog Cardiovasc Dis.

[2] Elliott P, Brown I (2007). Sodium intakes around the world.

[3] Korea Centers for Disease Control and Prevention (2013). Korea health statistics 2012: Korea National Health and Nutritional Examination Survey (KNHANES V-3).

[4] World Health Organization (2003). Diet, nutrition, and the prevention of chronic diseases: report of a joint WHO/FAO expert consultation.

[5] Kirkendall AM, Connor WE, Abboud F, Rastogi SP, Anderson TA, Fry M (1976). The effect of dietary sodium chloride on blood pressure, body fluids, electrolytes, renal function, and serum lipids of normotensive man. J Lab Clin Med.

[6] World Health Organization (2011). Strategies to monitor and evaluate population sodium consumption and sources of sodium in the diet.

[7] Espeland MA, Kumanyika S, Wilson AC, Reboussin DM, Easter L, Self M (2001). Statistical issues in analyzing 24-hour dietary recall and 24-hour urine collection data for sodium and potassium intakes. Am J Epidemiol.

[8] Sasaki S, Ishihara J, Tsugane S, JPHC (2003). Validity of a self-administered food frequency questionnaire in the 5-year follow-up survey of the JPHC Study Cohort I to assess sodium and potassium intake: comparison with dietary records and 24-hour urinary excretion level. J Epidemiol.

[9] Reinivuo H, Valsta LM, Laatikainen T, Tuomilehto J, Pietinen P (2006). Sodium in the Finnish diet: II trends in dietary sodium intake and comparison between intake and 24-h excretion of sodium. Eur J Clin Nutr.

[10] Shin EK, Lee HJ, Lee JJ, Ann MY, Son SM, Lee YK (2010). Estimation of sodium intake of adult female by 24-hour urine analysis, dietary records and dish frequency questionnaire (DFQ 55). Korean J Nutr.

[11] Rhodes DG, Murayi T, Clemens JC, Baer DJ, Sebastian RS, Moshfegh AJ (2013). The USDA Automated Multiple-Pass Method accurately assesses population sodium intakes. Am J Clin Nutr.

[12] Mattes RD, Donnelly D (1991). Relative contributions of dietary sodium sources. J Am Coll Nutr.

[13] Bull NL, Buss DH (1980). Contributions of foods to sodium intakes. Proc Nutr Soc.

[14] Tanaka T, Okamura T, Miura K, Kadowaki T, Ueshima H, Nakagawa H (2002). A simple method to estimate populational 24-h urinary sodium and potassium excretion using a casual urine specimen. J Hum Hypertens.

[15] Kawasaki T, Ueno M, Uezono K, Kawazoe N, Nakamuta S, Ueda K (1982). Average urinary excretion of sodium in 24 hours can be estimated from a spot-urine specimen. Jpn Circ J.

[16] Kawasaki T, Itoh K, Uezono K, Sasaki H (1993). A simple method for estimating 24 h urinary sodium and potassium excretion from second morning voiding urine specimen in adults. Clin Exp Pharmacol Physiol.

[17] Costa Ede A, Rose G, Klein CH, Achutti AC (1994). Diastolic pressure as an index of salt sensitivity. J Hum Hypertens.

[18] Mann SJ, Gerber LM (2010). Estimation of 24-hour sodium excretion from spot urine samples. J Clin Hypertens (Greenwich).

[19] Brown IJ, Dyer AR, Chan Q, Cogswell ME, Ueshima H, Stamler J (2013). Estimating 24-hour urinary sodium excretion from casual urinary sodium concentrations in Western populations: the INTERSALT study. Am J Epidemiol.

[20] Watson RL, Langford HG (1970). Usefulness of overnight urines in population groups. Pilot studies of sodium, potassium, and calcium excretion. Am J Clin Nutr.

[21] Liu K, Dyer AR, Cooper RS, Stamler R, Stamler J (1979). Can overnight urine replace 24-hour urine collection to asses salt intake?. Hypertension.

[22] Kamata K, Tochikubo O (2002). Estimation of 24-h urinary sodium excretion using lean body mass and overnight urine collected by a pipe-sampling method. J Hypertens.

