# Supplementary material for: Methodological issues in estimating sodium intake in the Korea National Health and Nutrition Examination Survey
Source: Epidemiol Health. 2014 Nov 28;36:e2014033. doi: 10.4178/epih/e2014033 (PMC4371387; doi:10.4178/epih/e2014033)
Supplement: Supplementary file 1 [file epih-36-e2014033-supplementary.pdf]

Introduction

과다한 나트륨 섭취는 고혈압, 심혈관계질환, 신장질환, 위암의 발생위험을 증가시키며, 간접적으로는 비만, 신장 결석 및 골다공증의 위험을 증가시키는 요인으로 작용한다. 나트륨 섭취량을 줄일 경우, 관련 만성질환의 유병률과 사망률이 크게 감소하는 것으로 보고되었다[1]. 우리나라는 전통적으로 김치, 장류, 젓갈류, 국·찌개 등의 섭취로 나트륨 섭취량이 높은 것으로 알려져 있어 나트륨 섭취를 줄이기 위한 정책적인 접근이 시급하다. 더 나아가, 정책적 감소목표를 설정하고 그 효과를 평가하기 위해서는 우선 우리나라 국민의 나트륨 섭취량을 정확하게 파악하고 그 추이를 지속적으로 모니터링 하는 것이 필요하다. 나트륨 섭취량은 24시간 회상법 (24-hour dietary recall)이나 식사일기법 (food record)을 이용한 영양조사 외에도 24시간 소변 (24-hour urine), 8-12시간 야간뇨 (overnight urine) 및 단회뇨 (spot urine)의 나트륨 배설량으로도 조사될 수 있는데, 각 나라마다 나트륨 섭취량을 조사하는 방법은 다양하며, 각기 장단점을 가지고 있다[2].

이에 본 원고에서는 우리나라 나트륨 섭취 현황을 파악하고 각 국가별 나트륨 섭취량 조사방법과 그 타당성을 고찰하고자 하며, 이를 통해 향후 우리나라 국민의 나트륨 섭취량과 섭취 추이를 보다 정확하게 파악할 수 있는 방법을 모색해보고자 한다.

한국인 나트륨 섭취 현황[3]

2012년 국민건강영양조사(Korea National Health and Nutrition Examination Survey) 결과, 한국인의 일일 나트륨 평균 섭취량은 4,546 mg (남자 5,212 mg, 여자 3,868 mg)으로 세계보건기구 (World health organization, WHO)의 일일 나트륨 권고상한치인[4] 2,000mg의 2배 이상을 섭취하는 것으로 나타났다(Figure. 1). 국민건강영양조사가 시작된 1998년 이후의 평균 나트륨 섭취량 (만 1세 이상, 연령표준화) 추이를 살펴보면, 1998년 4,582 mg에서 2005년 5,260 mg으로 증가하였으나, 개정 식품성분표가 적용된 2007년에 4,453 mg로 감소하였다. 동일한 식품성분표가 적용된 2007년부터의 추이를 살펴보면, 나트륨 섭취량은 2008년 4,608 mg, 2009년 4,618 mg, 2010년 4,785 mg로 다소 증가

추세였으나, 2011년 4,752 mg, 2012년 4,546 mg 으로 감소하였다. 남자는 식사량이 여자보다 많기 때문에 일일 나트륨 섭취량이 여자보다 약 1,000-1,500 mg 정도 높았으나, 연도별 추이는 남녀에서 비슷한 양상을 보였다.

2012년 국민건강영양조사에서 목표섭취량(9세 이상, 2,000 mg) 이상의 나트륨 과잉섭취 분율을 살펴본 결과(Figure. 2), 9세 이상 남자의 93.3%, 여자의 79.8%가 목표섭취량 이상을 섭취하고 있는 것으로 나타났으며, 30-40대의 과잉섭취 분율은 91.9%(남자 96.9%, 여자 86.7%)로 가장 높게 나타났다. 거주지역이나 소득수준에 상관없이 85% 이상의 대상자가 나트륨을 과잉섭취하고 있는 것으로 나타났다.

나트륨 섭취량 조사 방법의 장단점과 타당도

일일 나트륨 섭취량은 24시간 회상법, 식사일기법 등의 영양조사뿐만 아니라, 24시간 소변, 8-12시간 야간뇨, 단회뇨 등 소변 나트륨 배설량으로도 측정될 수 있다.

섭취한 나트륨의 85~95%는 소변으로 배설되고 소변 나트륨은 식품으로부터의 나트륨 섭취량과의 상관관계가 크다는 점에서 24시간 소변 분석법이 나트륨 섭취량을 측정하는데 가장 신뢰성 있는 방법으로 알려져 있다[5-6]. 그러나, 자유로운 생활을 하는 일반 인구집단에서 24시간 소변을 누락 없이 수집하는 것이 어렵고 참여자 부담이 크다는 단점이 있다. 영국의 National Diet and Nutrition Survey와 핀란드의 North Karelia Salt Project, FINMONICA Study, National FINRISK Study에서는 자국민의 평균 나트륨 섭취량을 파악하기 위해 국민 대표 표본 일부에서 24시간 소변을 수집하여 지속적으로 모니터링 하고 있다.

24시간 회상법이나 식사일기법을 이용한 영양조사는 대규모 인구집단 조사에서 비교적 용이하게 수행할 수 있는 방법이나, 지난 1일 동안 섭취한 모든 음식의 종류와 그 양을 정확하게 기억하거나 조리 시 첨가한 소금의 양을 정확하게 파악하기 어렵고, 자료 처리 시 음식의 표준 식품재료량을 적용할 경우 개인별 소금 섭취량 차이가 반영되지 않는 단점을 가진다. 또한, 식품성분표(food composition table)가 5년 주기로 개정되므로 년도별 추이 비교에 제한점을 가진다. 우리나라의 국민건강영양조사는 1일간

24시간 회상법, 미국의 국가건강영양조사(National Health and Nutrition Examination Survey)는 1-2일 간 24시간 회상법, 일본의 국가영양조사(Japanese National Nutrition Survey)는 1일 간(1995년 이전에는 3일 간) 반실측량 식사기록법(semi-weighted food record method)을 이용하여 나트륨 섭취량을 산출하고 있다. 24시간 식이회상 자료를 이용하여 나트륨 섭취량을 산출하는 방법의 타당도는 각 국가별 주요 섭취 음식과 조리 특성에 따라 다른 것으로 나타났다 (Table 1)[7-11]. 서구 지역은 나트륨 섭취의 주요 공급원이 가공식품 (미국 77%[12], 영국 65-70%[13])으로 식품 섭취량만으로도 개인별 나트륨 섭취의 상당량을 파악할 수 있어 24시간 소변과의 상관성이 0.3~0.4 정도인 반면[7, 9, 11], 한국인 대상 연구 결과에서는 24시간 회상법을 이용한 나트륨 섭취량과 24시간 소변 나트륨 간 상관성이 0.11로 매우 낮았다[10]. 이와 같이 낮은 상관성을 보이는 이유 중 하나는 한국인의 소금 섭취 주요 공급원이 김치, 국, 찌개 등으로 개인 간 소금 염미도와 조리 시 첨가량에 따라 차이가 클 수 있음에도 불구하고 조리한 음식의 식품재료량과 첨가한 조미료량을 파악하지 못하는 경우, 음식의 표준 식품재료량을 적용하여 개인의 나트륨 섭취량 차이를 파악하기 어렵기 때문인 것으로 사료된다.

단회뇨(spot urine)의 나트륨 농도를 이용하여 일일 나트륨 섭취량을 추정하는 방법은 참여자 부담이 적은 장점이 있지만, 나트륨 농도는 수분 섭취량에 영향을 받기 쉬우므로 개인별 정확한 나트륨 섭취량을 파악하기에는 한계가 있다. 이러한 단점에도 불구하고, 단회뇨를 이용한 나트륨 섭취의 집단 평균값은 집단 간 비교 및 특정 집단의 연도별 추이를 파악하는데 유용한 방법으로 제안되었다[14]. 국외 연구에서 단회뇨의 나트륨 농도는 24시간 소변 나트륨 농도와의 상관성이 0.28-0.86[14-19]으로, 단회뇨 수집 시간과 횟수에 따라 차이를 보였다 (Table 2). 밤사이 8-12시간 야간뇨(overnight urine)를 이용하는 방법은 24시간 소변보다는 수집이 용이한 장점을 가지는 반면, 시간을 정확하게 지켜 완전하게 수집해야 하고 낮과 밤 소변 분비의 항상성 가정이 필요하다는 제한점을 가진다. 야간뇨의 경우, 24시간 소변 나트륨 배설량과 상관성이 0.59-0.78[20-22]로, 단회뇨 보다 24시간 소변 대체 방법으로 더 타당한 것으로 보고되었다 (Table 2).

국민건강영양조사에서 나트륨 섭취 조사 방법 개선방안

국민건강영양조사에서는 24 시간 회상법을 이용하여 우리나라 국민의 평균 일일 나트륨 섭취량을 추정하고 있으나, 앞서 기술한 바와 같이 24 시간 회상법은 그 조사방법과 자료처리 방법이 가지는 고유한 특성으로 나트륨 섭취량을 정확하게 파악할 수 없는 제한점이 있다. 이에, 영양조사의 제한점을 보완하기 위해 국민건강영양조사에서는 소변을 이용하여 나트륨 섭취량을 추정하는 방법을 도입하고자 하며, 도입에 앞서 소변 나트륨 측정 방법의 실현가능성과 타당성 검증이 필요하다. 이에 「국민건강영양조사 나트륨 섭취 추정방법 연구(Study on estimation methods of sodium intake in the Korea National Health and Nutrition Examination Survey)」를 질병관리본부(Korea Centers for Disease Control and Prevention)의 정책연구용역과제로 현재 진행 중이며, 국민건강영양조사 참여자 중 약 300 명의 24 시간 소변, 8 시간 야간뇨, 단회뇨를 수집하여 우리나라 고유의 나트륨 섭취량 추정식을 개발하고 24 시간 소변 나트륨 총배설량과의 비교를 통해 단회뇨와 8 시간 야간뇨를 이용한 나트륨 섭취 추정방법의 타당성을 검증할 계획이다. 이 연구를 통하여 우리나라 국민의 나트륨 섭취량 추정에 가장 적절한 소변수집 방법, 소변수집 시간 및 수집량에 대한 지침을 마련하여 국민건강영양조사에 도입할 계획이다. 단회뇨나 8 시간 야간뇨로 추정된 나트륨 섭취량은 국민건강영양조사의 24 시간 회상법을 이용한 영양조사 결과를 보완할 수 있을 것으로 기대되며, 이를 통해 추정된 우리나라 국민의 나트륨 섭취량은 향후 나트륨 감소정책 추진과 보건정책 수립 및 효과 평가에 필요한 기초자료로 활용될 수 있을 것으로 기대된다.

Acknowledgement

The KNHANES has been financially supported by the Health Promotion Fund with administrative support by the Ministry of Health and Welfare.

References

1. He FJ, MacGregor GA. Reducing population salt intake worldwide: From evidence to implementation. Prog Cardiovasc Dis 2010; 52: 363-82.
2. Elliott P and Brown I. Sodium intakes around the world. Geneva, Switzerland: World Health Organization Press; 2006.
3. Korea Centers for Disease Control and Prevention. Korea Health Statistics 2012 : Korea National Health and Nutrition Examination Survey (KNHANES V-3). Seoul, Korea: Korea Ministry of Health and Welfare; 2013.
4. World Health Organization. Diet, nutrition and the prevention of chronic diseases (WHO Technical report series, No. 916). Geneva, Switzerland: World Health Organization Press; 2003.
5. Kirkendall AM, Connor WE, Abboud F, Rastogi SP, Anderson TA, Fry M. The effect of dietary sodium chloride on blood pressure, body fluids, electrolytes, renal function, and serum lipids of normotensive man. J Lab Clin Med 1976; 87(3): 411-34.
6. World Health Organization. Strategies to monitor and evaluate population sodium consumption and sources of sodium in the diet. Geneva, Switzerland: World Health Organization Press; 2011.
7. Espeland MA, Kumanyika S, Wilson AC, Reboussin DM, Easter L, Self M, et al. Statistical issues in analyzing 24-hour dietary recall and 24-hour urine collection data for sodium and potassium intakes. Am J Epidemiol 2001; 153(10): 996-1006.
8. Sasaki S, Ishihara J, Tsugane S, JPHC. Validity of a self-administered food frequency questionnaire in the 5-year follow-up survey of the JPHC Study Cohort I to assess sodium and potassium intake: comparison with dietary records and 24-hour urinary excretion level. J Epidemiol 2003; 13(1 Suppl): S102-5.
9. Reinivuo H, Valsta LM, Laatikainen T, Tuomilehto J, Pietinen P. Sodium in the Finnish diet: II Trends in dietary sodium intake and comparison between intake and 24-h excretion of sodium. Eur J Clin Nutr 2006; 60(10): 1160-7.
10. Shin EK, Lee HJ, Lee JJ, Ann MY, Son SM, Lee YK. Estimation of Sodium Intake of Adult Female by 24-Hour Urine Analysis, Dietary Records and Dish Frequency Questionnaire (DFQ 55). Korean J Nutr 2010; 43(1): 79 ~85.
11. Rhodes DG, Murayi T, Clemens JC, Baer DJ, Sebastian RS, Moshfegh AJ. The USDA Automated Multiple-Pass Method accurately assesses population sodium intakes. Am J Clin Nutr 2013; 97(5): 958-64.

12. Mattes RD, Donnelly D. Relative contributions of dietary sodium sources. J Am Coll Nutr 1991; 10(4): 383-93.

13. British Nutrition Foundation. Salt in the Diet. London, UK: British Nutrition Foundation; 1994.

14. Tanaka T, Okamura T, Miura K, Kadowaki T, Ueshima H, Nakagawa H, et al. A simple method to estimate populational 24-h urinary sodium and potassium excretion using a casual urine specimen. J Hum Hypertens 2002; 16(2): 97-103.

15. Kawasaki T, Ueno M, Uezono K, Kawazoe N, Nakamuta S, Ueda K, et al. Average urinary excretion of sodium in 24 hours can be estimated from a spot-urine specimen. Jpn Circ J 1982; 46(9): 948-53.

16. Kawasaki T, Itoh K, Uezono K, Sasaki H. A simple method for estimating 24 h urinary sodium and potassium excretion from second morning voiding urine specimen in adults. Clin Exp Pharmacol Physiol 1993; 20(1): 7-14.

17. Costa Ede A, Rose G, Klein CH, Achutti AC. Diastolic pressure as an index of salt sensitivity. J Hum Hypertens 1994; 8(9): 703-9.

18. Mann SJ, Gerber LM. Estimation of 24-hour sodium excretion from spot urine samples. J Clin Hypertens 2010; 12(3): 174-80.

19. Brown IJ, Dyer AR, Chan Q, Cogswell ME, Ueshima H, Stamler J, et al. Estimating 24-hour urinary sodium excretion from casual urinary sodium concentrations in Western populations: the INTERSALT study. Am J Epidemiol 2013; 177(11): 1180-92.

20. Watson RL, Langford HG. Usefulness of overnight urines in population groups. Pilot studies of sodium, potassium, and calcium excretion. Am J Clin Nutr 1970; 23(3): 290-304.

21. Liu K, Dyer AR, Cooper RS, Stamler R, Stamler J. Can overnight urine replace 24-hour urine collection to assess salt intake? Hypertension 1979; 1(5): 529-36.

22. Kamata K, Tochikubo O. Estimation of 24-h urinary sodium excretion using lean body mass and overnight urine collected by a pipe-sampling method. J Hypertens 2002; 20(11): 2191-7.
